# Lung Metastatic Nodules as First Presentation of Synovial Cardiac Sarcoma

**DOI:** 10.1186/1749-8090-10-S1-A327

**Published:** 2015-12-16

**Authors:** F Ampatzidou, T Karaiskos, K Vasiliadis, A Cheva, CP Koutsogiannidis, A Madesis, M Sileli, G Drossos

**Affiliations:** 1 Cardiothoracic Surgery Department, G. Papanikolaou Hospital, Thessaloniki, 57010, Greece; 2 Cardiology Department, G. Papanikolaou Hospital, Thessaloniki, 57010, Greece; 3 Histopathology Department, G. Papanikolaou Hospital, Thessaloniki, 57010, Greece

## Background/Introduction

Synovial sarcomas are rare tumors accounting for approximately 1% from all cardiac neoplasms and present with a variety of symptoms depending on their location and size.

## Aims/Objectives

To describe a unique case of a 36 year old male presenting with bilaterally pulmonary nodules as a result of right heart synovial sarcoma.

## Method

**Figure 1:**
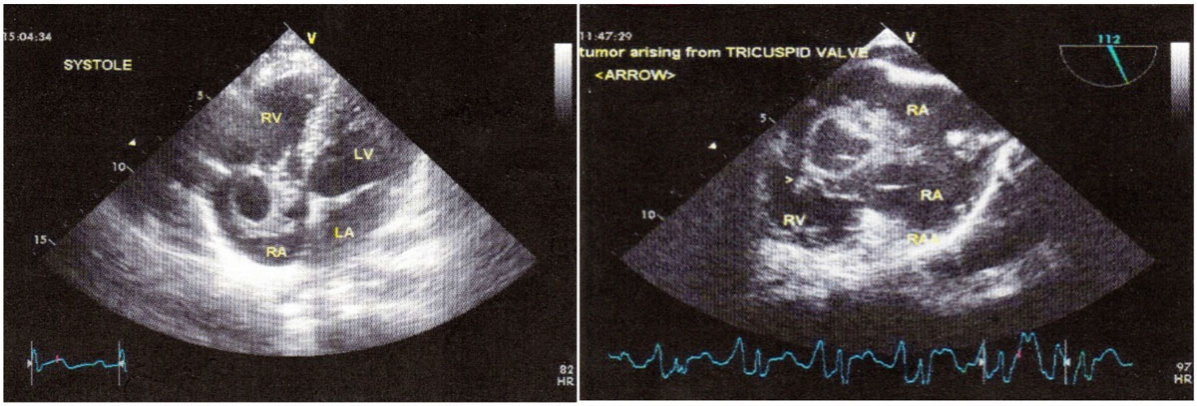
**TTE-TEE Echocardiography: Right atrial mass**.

**Figure 2:**
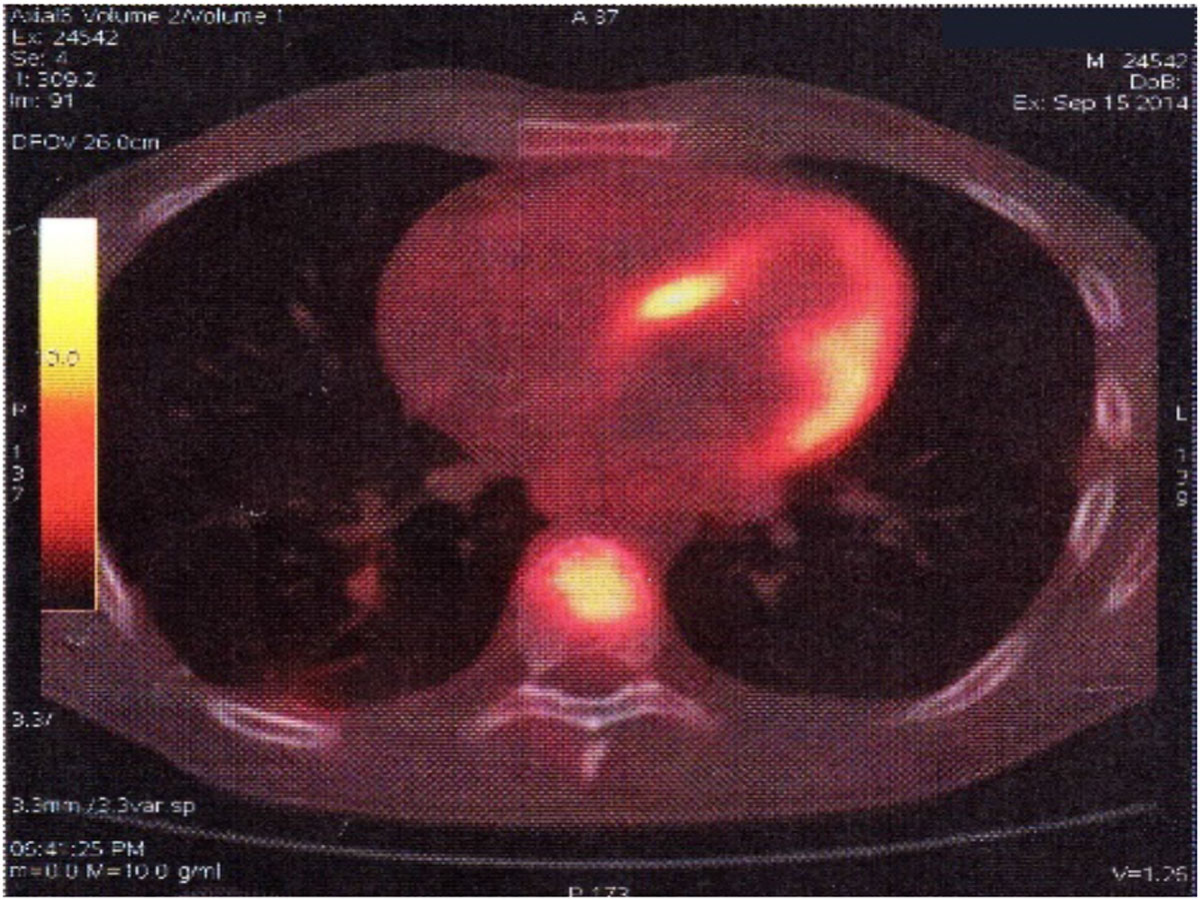
**18F PET-CT scan**. Lytic lesionin thoracic vertebrae body.

Video-assisted thoracoscopic biopsy (due to newly diagnosed pulmonary nodules) revealed mesenchymal malignant neoplasm. Echocardiography showed a large mass located in the right atrium, arising from the anterior tricuspid valve. [Fig 1] Magnetic resonance imaging (MRI) of the heart demonstrated further anatomic details.18F PET-CT scan revealed increased tracer uptake in lung nodule and in lytic lesion of the 8th thoracic vertebrae body indicating a spinal cord metastasis [Fig 2].

## Results

**Figure 3:**
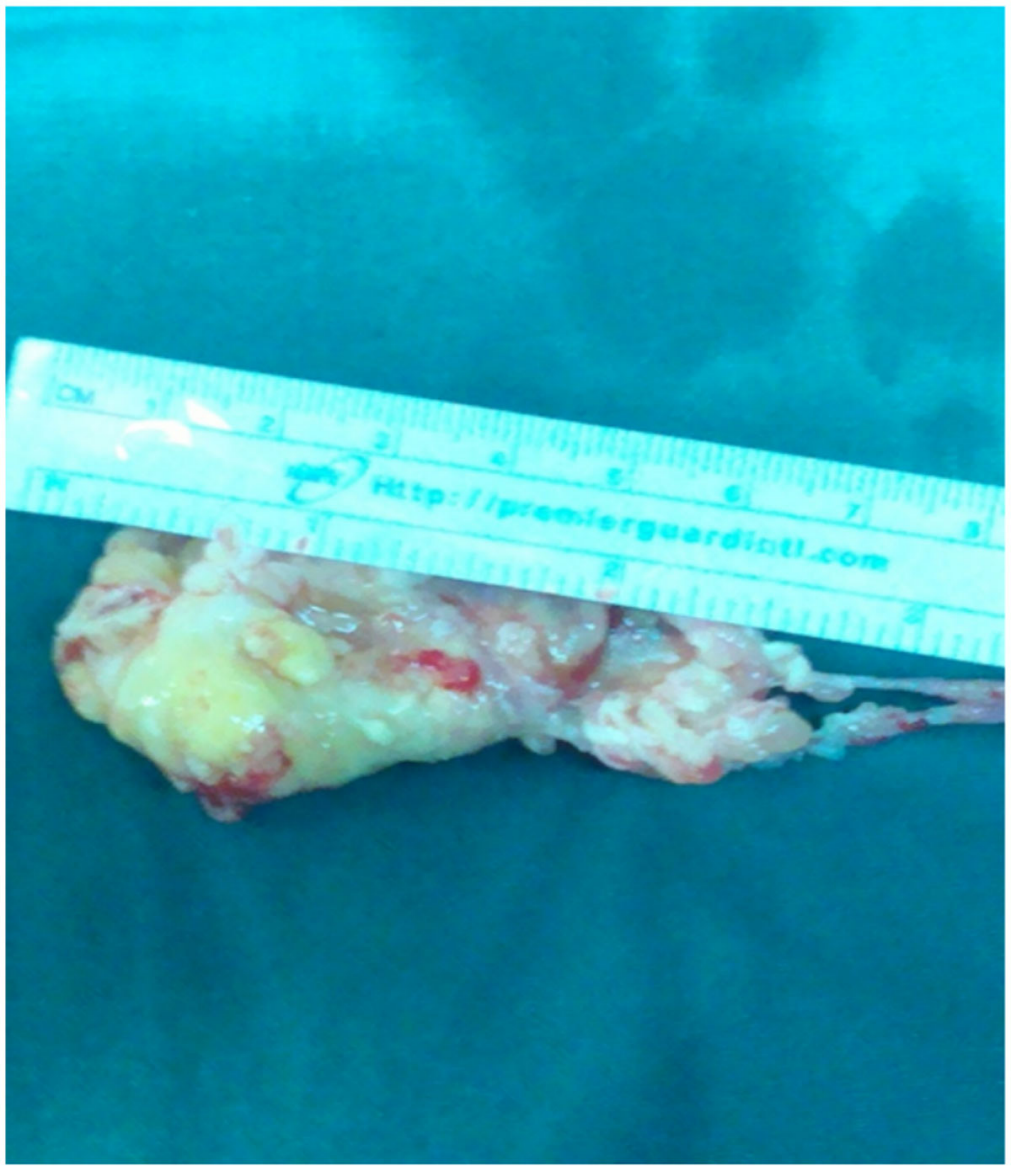
**Excised mass, 6 cm long**.

The patient underwent median sternotomy and aorto-bicaval cannulation. After longitudinally opening of the right atrium, a 6-cm long pedunculated tumor originating from the atrial wall and part of the septal cusp of the tricuspid valve was identified. The tumor was completely resected with part of the invaded cusp. [Fig 3] The septal cusp was repaired with pericardial patch and tricuspid valve repair was completed with the placement of annuloplasty ring. The left lower lobe nodule was excised with wedge resection, while the nodules of the left upper lobe and the right lower lobe were ablated with a radiofrequency device because of their central location. The patient was discharged uneventfully on the 6th postoperative day.

**Figure 4:**
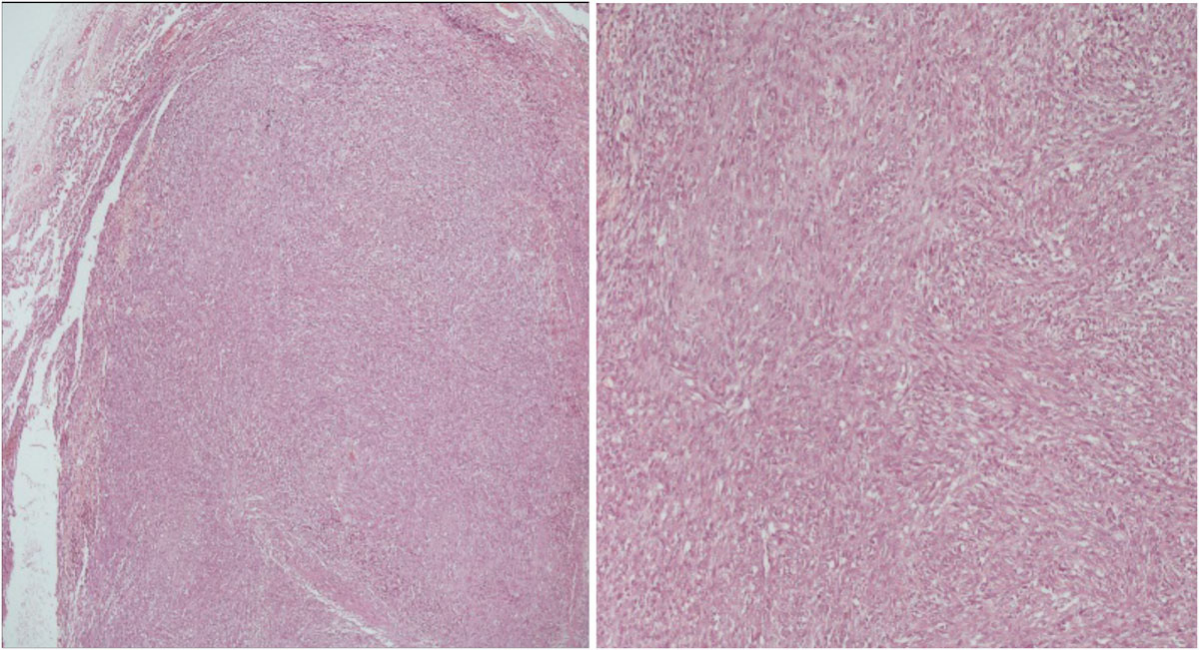
**Histopathologic image: Monophasic synovial sarcoma**. *Hematoxylin*-eosin staining of tissue showing the characteristic infiltration by spindle-like cells with dense arrangement and large nuclei.

Histopathologic testing results, showed neoplastic infiltration by spindle-like cells with dense arrangement, large nuclei, atypia, polymorphism and increased mitotic rate. Findings were indicative of monophasic synovial sarcoma mainly from spindle cells [Fig 4].

## Discussion/Conclusion

We present a unique case of a patient with synovial sarcoma with pulmonary nodules as first manifestation.

## Consent

Written informed consent was obtained from the patient for publication of this abstract and any accompanying images. A copy of the written consent is available for review by the Editor of this journal.

